# The Indirect Costs of Late-Life Depression in the United States: A Literature Review and Perspective

**DOI:** 10.3390/geriatrics1040030

**Published:** 2016-11-14

**Authors:** Caitlin E. Snow, Robert C. Abrams

**Affiliations:** Department of Psychiatry, NewYork-Presbyterian Hospital/Weill Cornell Medicine, 525 E 68th St, New York, NY 10065, USA; rabrams@med.cornell.edu

**Keywords:** late-life depression, geriatric depression, indirect costs, economic burden, cost-of-illness, caregiver burden

## Abstract

Late-life depression is a leading cause of disability in older adults and is associated with significant economic burden. This article draws from the existing literature and publicly available databases to describe the relative importance of the indirect costs associated with late-life depression. The authors found that unpaid caregiver costs represent the largest component of the indirect costs of late-life depression, with the highest level of economic burden attributed to the majority of care recipients who have fewer depressive symptoms. Other indirect costs, such as productivity losses related to early retirement, reduced ability to fulfill work and family functions and diminished financial success were mostly under-appreciated in the literature. Also, mortality cost estimates provided little clarity, employing variable methodologies and revealing mixed results. With respect to late-life suicide studies, studies approximated both economic costs and savings. More rigorous efforts to evaluate the indirect costs of late-life depression would afford a better understanding of the social and economic toll of this disorder and could influence the allocation of resources for research and treatment.

## 1. Introduction

Late-life depressive disorders are prevalent and associated with substantial economic burden. Approximately 10%–15% of older persons in primary care settings suffer from a depressive illness [1,2], a condition which has been shown to confer significant morbidity and mortality [3]. Depressive symptoms in the elderly have been associated with functional decline [4], executive dysfunction [5], diminished health-related quality of life [6] and higher utilization of primary healthcare services [7,8,9]. Although older adults frequently seek help for mental health issues from a primary care provider [10], non-specialist physicians tend to under-recognize [11,12] and under-treat [13,14] geriatric depression.

As the population of older adults in the United States expands, it is possible, if not likely, that the total costs associated with late-life depression will increase as well. Estimates of the economic burden of a disease to society as a whole have relied on cost-of-illness studies that employ a wide range of methodologies. The principal aim of these cost-of-illness studies is to inform resource allocation by policymakers. Numerous reports have demonstrated high direct costs associated with late-life depression, arising mainly from the increased utilization of medical resources across healthcare settings (outpatient, inpatient, emergency room, nursing home); other direct costs include expenses for pharmaceuticals and related but non-medical resources (social services, transportation) [15,16,17]. However, the indirect costs of late-life depression have received little attention. This article relies on findings from the literature and publicly available databases to describe the relative importance of the indirect costs associated with late-life depression, and to identify the areas of highest cost burden. 

## 2. Indirect Costs of Late-Life Depression

Indirect costs of late-life depression are defined as productivity losses due to illness-associated morbidity and mortality that are endured by the individual, family, employer, and society [18]. Indirect costs are typically not registered in measurable monetary transactions, and therefore can be difficult to approximate. Reflecting the particular social and medical circumstances of aging persons, the indirect costs associated with late-life depression are distinct from those associated with depressed younger adults, and for this reason cannot be accurately extrapolated from mixed-age data. For example, indirect costs associated with late-life depression may be reflected in the relationship with retirement, a bidirectional exchange in which depression may contribute to early retirement, and in the other direction, retirement itself may operate as an etiological stressor for depression [19]. Other sources of indirect costs might include such productivity losses as more days out of household roles, diminished financial success, and increased need for informal caregiving provided by unpaid family and friends. Indirect costs of late-life depression are summarized in Table 1.

## 3. Methods

The authors conducted a comprehensive literature review of peer-reviewed journal articles published in English using the databases PubMed, Web of Science, Google Scholar, and CDC.gov. The search was conducted without time limit, and the date last searched was 9 July 2016. Bibliographies of the articles obtained in the initial search were examined to identify additional relevant studies. Combined searches using the following terms were performed: “cost” or “economic burden” or “cost-of-illness” or “indirect cost” or “caregiver burden” or “mortality costs” or “productivity loss” and “late-life” or “geriatric” or “elderly” or “older adult” and “depression” or “depressive disorder”. To be included in the review, the selected studies were required to present a methodology section sufficient for evaluation and to include a monetary estimate of an indirect cost of late-life depression. Publications were excluded if they did not address late-life depression, if they assessed the burden of late-life depression in non-monetary terms, if they focused only on direct costs, or if they were derived from a non-United States population. Costs in all studies were reported in US dollars and then inflated to the year 2016.

## 4. Results

The combined search generated 1480 non-duplicate journal articles. Of these, 1436 records were excluded after initial screen of abstracts and 44 full texts were assessed for eligibility. Thirty-eight articles were excluded because they did not provide a monetary estimate of the indirect cost of late-life depression, and two articles were excluded because they were derived from a non-United States population. Only four articles provided a monetary estimate of an indirect cost associated with late-life depression in the United States (see Table 2), with the remaining articles providing qualitative support.

## 5. Morbidity Costs

### 5.1. Workplace and Functional Impairment Costs

Few articles specifically examine productivity losses associated with late-life depression such as those attributable to early retirement [24], reduced ability to fulfill work and family functions, and diminished financial success [25,26]. In an older population, productivity losses are often manifested in reduced ability to participate in family functions such as caregiving of other older adults or grandchildren and in diminished financial success generally. The functional impairment and disability that accompany late-life depression are important contributors to the reduction in older adults’ productivity in these areas [4]. The 2015 national statistics revealed that more than 50% of caregivers are aged 50 and above, and 19% of caregivers are aged 65 and above [27]. Because many families rely on older adults to fulfill a range of family functions, it is likely that productivity losses in late life represent a significant economic burden to society. However, approximations of the monetary value are typically excluded in the literature.

Productivity losses among older adults may be under-estimated in part due to methodological barriers. Cost-of-illness studies most often employ the human capital method as a proxy for estimating a healthy individual’s future contributions to a society. To the extent that these future contributions are sacrificed by illness or disability, the human capital method offers an indirect measure of productivity losses [18]. This approach approximates unrealized workplace earnings by combining average wage-based earnings with work time lost due to illness [28]. However, human capital measures are typically limited to the working-age population and exclude productivity losses of adults over the retirement age. The full retirement age in the United States is increasing [29], and assuming that other factors are unchanged, a rising retirement age should result in higher levels of depression-related workplace productivity losses among older adults than have been previously accounted for using human capital methodology.

### 5.2. Caregiving Costs

The cost burden of depressed individuals’ family members is significant. Depressive symptoms adversely impact older adults’ independent functioning, and are consequently associated with increased levels of unpaid caregiving from family and friends [19]. In fact, caregiving requirements have been shown to rise with increasing depression severity of the care recipient [20]. One prevalence study employed an opportunity cost estimate approach to assign an economic value to informal caregiving costs associated with late-life depression [20]. After controlling for chronic comorbid medical conditions and socio-demographic factors, the authors estimated the yearly cost of unpaid caregiving for national survey respondents in each of three depressive symptom categories by multiplying the 2000 median national wage for a home health aide by the adjusted hours of caregiving. A national prevalence estimate of depressive symptoms from the AHEAD study [30] was then used to approximate the yearly national cost of informal caregiving associated with depressive symptoms in older Americans. Using that method the authors arrived at an estimate of additional yearly caregiving costs totaling approximately $9.1 billion ($12.7 billion in year-2016 dollars using the relevant US government Consumer Price Index (CPI) data [31]). A cost breakdown reveals $5 billion ($7 billion in year-2016 costs) in caregiver costs for individuals with 1–3 depressive symptoms and $4.1 billion ($5.7 billion in year-2016 costs) in caregiver costs for individuals with 4–8 depressive symptoms [20].

However, the cost of $12.7 billion year-2016 dollars estimated by Langa et al. [20] for unpaid caregiving of elderly depressives is understated as a result of conservative measures and low-range opportunity cost estimates. Additionally, the possible downstream economic effects of caregiving, including the negative health consequences and productivity losses associated with caregiver burden [32], were not considered. Family involvement in the care of depressed older adults can both promote and impede good outcomes, and therefore provides unclear economic cost benefit [33].

## 6. Mortality Costs

There are several studies that estimate the cost of suicide, using the general approach of combining the direct costs of medical expenditures with the indirect costs of the net present value of productivity losses [21,22,23] (see Table 2). The Centers for Disease Control and Prevention (CDC) maintains the Web-based Injury Statistics Query and Reporting System (WISQARS), an online interactive database that tabulates reported deaths by suicide in the United States and estimates associated costs. According to WISQARS, the 2010 national cost of self-inflicted fatal injuries in individuals aged 65 and above was approximately $1,130,688,000 [22]. The direct medical costs represent only a 1.8% contribution to these overall costs, with indirect work loss costs accounting for the remainder. On the basis of prior literature, 50% of suicides can be conservatively attributed to depression [26,34]. This figure yields a suicide-related indirect costs estimate associated with late-life depression of $555,014,000 ($611,468,354 year-2016 dollars). Of note, a higher national cost of suicide in older adults was estimated in a recent study [23] by adjusting the methodology of prior studies [21,22,35] to account for an increased rate of suicide, under-reporting of suicides, and increased direct costs due to higher levels of medical intervention. 

These calculations should be interpreted with caution because of their several limitations. Most notably, the cost estimates are based on an assumption of average productivity across different demographics, and do not subtract for the diminished productivity of depressed individuals. In fact, suicide as an outcome of late-life depression may result in economic savings for a society via the avoidance of future illness-related direct and indirect costs [35]. This purely economic perspective must be adjusted to real-life clinical situations, in which such factors as the psychological suffering of family members and promotion of life-prolonging interventions also exert influence.

## 7. Discussion

This article delineates the indirect costs associated with late-life depression as described in the medical literature. Unpaid caregiver costs represent the largest component of the indirect costs, with the highest level of economic burden attributed to the majority of care recipients who have fewer or diagnostically subthreshold depressive symptoms. Work loss productivity costs in depressed older adults are under-appreciated, particularly in the “young old” population (aged 65–74) in which many individuals remain engaged in the workforce. Finally, mortality cost estimations reveal mixed results, with studies approximating both economic costs and savings associated with late-life suicide [21,22,23,35].

There are several limitations to this analysis. First, studies that include estimations of indirect costs associated with late-life depression are scarce, and those available use varying methodologies, thereby confounding the tasks of comparison and interpretation. Furthermore, of the studies that do include indirect cost estimations, the calculations mostly neglect the value of leisure. A methodological approach that incorporates the intrinsic value of life, such as a willingness-to-pay approach, may provide a better approximation of the morbidity costs of late-life depression [18].

## 8. Conclusions

The analysis in this article suggests that caregiver costs represent the largest component of the indirect costs associated with depression in older adults, in contrast to findings in the general population in which workplace costs represent the largest component of the indirect costs of depression [26]. Further research is needed to better approximate the indirect costs associated with late-life depression. In addition, studies should compare the costs of depression across the lifespan so as to inform resource allocation according to age group.

## Figures and Tables

**Table 1:** Indirect costs of late-life depression.

Morbidity, e.g., Productivity Losses
Workplace costs Absenteeism (missed days of work) Presenteeism (reduced productivity at work) Early retirement
Functional impairment costs Reduced ability to fulfill family functions and needs (household productivity) Poor financial decision-making
Unpaid caregiving costs
**Mortality, e.g., Suicide** Future productivity lost

**Table 2:** Monetary estimates of indirect costs of late-life depression in the United States.

Study	Indirect Cost	Age (sample size)	Direct Costs (Year-2016 Dollars)	Indirect Costs (Year-2016 Dollars)
Langa et al. (2004) [20]	Informal caregiving	70+ (6,649)	N/A	$9.1 billion ($12.7 billion)
Corso et al. (2000) [21]	Suicide	65+ (5,319)	$15 million ($21 million)	$630 million ($879 million)
CDC WISQARS (2010) [22]	Suicide	65+ (5,994)	$20.7 million ($22.8 million)	$1.11 billion ($1.21 billion)
Shepard et al. (2013) [23]	Suicide	65+ (12,036 *)	$1.74 billion ($1.81 billion) **	

* Calculated as reported number of suicides + adjustment for under-reporting in age categories 65–74 and 74+. ** Combined direct and indirect costs.
